# Population genetics and geographic origins of mallards harvested in northwestern Ohio

**DOI:** 10.1371/journal.pone.0282874

**Published:** 2023-03-15

**Authors:** Michael L. Schummer, John Simpson, Brendan Shirkey, Samuel R. Kucia, Philip Lavretsky, Douglas C. Tozer

**Affiliations:** 1 Department of Environmental Biology, State University of New York College of Environmental Science and Forestry, Syracuse, New York, United States of America; 2 Winous Point Marsh Conservancy, Port Clinton, Ohio, United States of America; 3 Department of Biological Sciences, University of Texas at El Paso, El Paso, Texas, United States of America; 4 Long Point Waterfowl and Wetlands Research Program, Birds Canada, Port Rowan, Ontario, Canada; National Cheng Kung University, TAIWAN

## Abstract

The genetic composition of mallards in eastern North America has been changed by release of domestically-raised, game-farm mallards to supplement wild populations for hunting. We sampled 296 hatch-year mallards harvested in northwestern Ohio, October–December 2019. The aim was to determine their genetic ancestry and geographic origin to understand the geographic extent of game-farm mallard introgression into wild populations in more westward regions of North America. We used molecular analysis to detect that 35% of samples were pure wild mallard, 12% were early generation hybrids between wild and game-farm mallards (i.e., F1–F3), and the remaining 53% of samples were assigned as part of a hybrid swarm. Percentage of individuals in our study with some form of hybridization with game-farm mallard (65%) was greater than previously detected farther south in the mid-continent (~4%), but less than the Atlantic coast of North America (~ 92%). Stable isotope analysis using *δ*^2^H_f_ suggested that pure wild mallards originated from areas farther north and west than hybrid mallards. More specifically, 17% of all Ohio samples had *δ*^2^H_f_ consistent with more western origins in the prairies, parkland, or boreal regions of the mid-continent of North America, with 55%, 35%, and 10% of these being genetically wild, hybrid swarm, and F3, respectively. We conclude that continued game-farm introgression into wild mallards is not isolated to the eastern population of mallards in North America, and may be increasing and more widespread than previously detected. Mallards in our study had greater incidence of game-farm hybridization than other locales in the mid-continent but less than eastern North American regions suggesting further need to understand game-farm mallard genetic variation and movement across the continent.

## Introduction

Humans and mallards (*Anas platyrhynchos*) have a closely linked history since their domestication in China around 500 B. C. [1, 2]. Nearly all domestic ducks (e.g., Pekin duck) are decedents of wild, Eurasian mallards [1]. The intentional supplementation of wild populations is common practice for many game species [3–6] including waterfowl [7, 8]. Although wild mallard populations naturally have a Holartic distribution, the intentional introduction of wild and domesticated forms has artificially increased their distribution to nearly everywhere but the Poles [9]. Among domestic mallards, the intentional release of the game-farm mallard breed is responsible for the majority of their range expansion. Started in the early 20^th^ century, the release of game-farm mallards continues intensively today in Europe (6-million annually) [10, 11] and eastern North America (≥ 200,000 annually) [12–14] largely for the supplementation of wild mallard populations for hunting purposes [14, 15]. Much like other domesticated mallard forms, all game-farm mallards have a Eurasian origin, and those being released in Europe and North America are from the same genetic source [10–12]. Introduction of game-farm mallards were thought to have no impact on wild populations, but only recently have molecular studies begun to estimate levels of introgression of game-farm genes into wild mallard populations. Wild mallards are genetically panmictic [16], but release of game-farm mallards has changed the genetic ancestry of wild populations in Europe [17] and North America [18, 19]. Increasing prevalence of game-farm genes within wild populations is predicted to decrease mallard fitness because of introduction of artificially selected, maladaptive traits [17, 19], and this has been hypothesized to at least partially explain recent mallard population declines in eastern North America [19] and central Europe [17].

In North America, studies continue to find a close association among game-farm mallard ancestry prevalence and regions of intense game-farm mallard release programs [14, 19]. Among regions, > 90% of releases of game-farm mallards have occurred along the Atlantic coast of North America: ~500,000 game-farm mallards were released annually from 1920–1950 [13], and ≥ 200,000 have been released annually since, although these numbers are not well-documented [14, 15]. Elsewhere in North America release of game-farm mallards were at a smaller scale (e.g., 16,983 released as part of population supplementation programs in Ohio from 1959–1963) [20]. By 2010, only ~8% of mallards on the Atlantic coast were pure wild [18] and the prevalence of game-farm × wild mallard hybrids generally decreases westward [18], but finer scale studies of mallard genetics are needed to understand the movement and spatial distribution of game-farm × wild mallard hybrids. Specifically, Lavretsky et al. [18] detected that ~40% of mallards sampled throughout the Mississippi flyway of North America had substantial game-farm mallard introgression, whereas southern parts of the same flyway only reported ~4% as hybrids [21]. Thus, the distribution of and mechanisms for game-farm mallard ancestry throughout North America remains a critical information gap.

**Fig 1:**
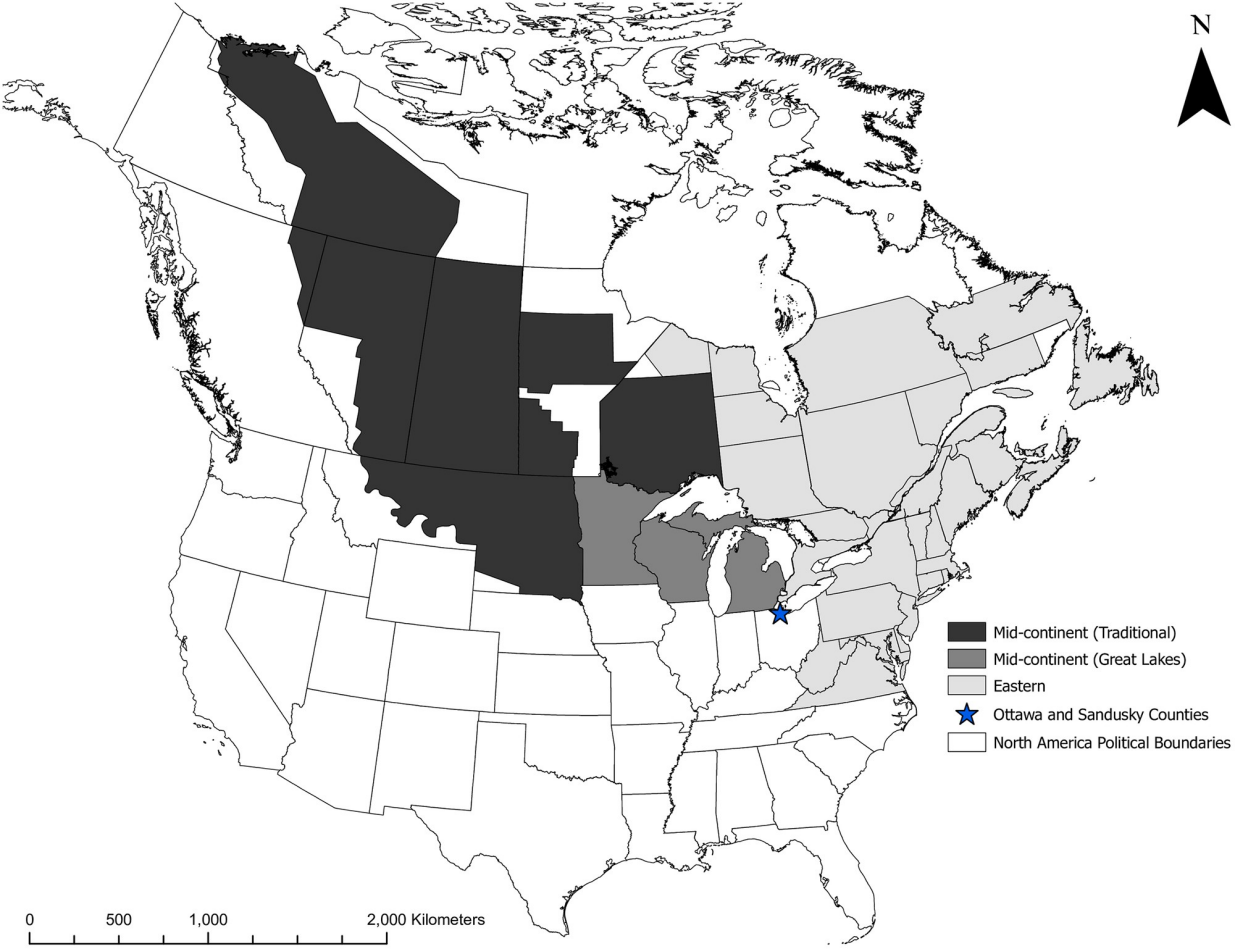
Waterfowl survey areas currently assigned to the eastern and mid-continent stocks of mallards as identified by the United States Fish and Wildlife Service (USFWS) for management purposes. Spatial layers of populations were provided by the USFWS and base outline political boundary layers are free, open-source from ESRI. WBPHS, Eastern Survey, and Great Lakes shapefiles were provided by USFWS. U.S. political boundaries were obtained through the U.S. Census Bureau (https://www.census.gov/geographies/mapping-files/time-series/geo/carto-boundary-file.html). Canadian province political boundaries were obtained through Open Canada census Bureau (https://open.canada.ca/data/en/dataset/a883eb14-0c0e-45c4-b8c4-b54c4a819edb). Except for the shapefiles listed above, no external data was used for the creation of the figure. ESRI ArcMap 10.8 was used for the creation of the figure.

Mallards in North America are managed as western, mid-continent, and eastern breeding populations (Fig 1). Among these, the eastern mallard population steadily increased until 1999, with populations declining thereafter by 40% [22, 23]. However, observed declines were unequal across their eastern range [23]. The Atlantic Flyway Breeding Waterfowl Plot Survey (AFBWPS), which occurs from Virginia to New Hampshire, identified mallard population declines of ~50% (880,000 in 1999 to 448,500 in 2017), whereas the eastern survey area of the Waterfowl Breeding Population and Habitat Survey (WBPHS) that includes eastern Canada and Maine suggested stable to slightly increasing abundance from 171,000 to 204,200 during the same time period [23]. The combined population estimates from the AFBWPS and WBPHS are used to monitor the eastern mallard population and suggest an ~ 38% decline. The mid-continent population of mallards is described as those in the traditional WBPHS area (strata 13–18, 20–50, and 75–77) and the Great Lakes (Michigan, Minnesota, and Wisconsin) (Fig 1). The mallard population in the traditional WBPHS area increased by 75% from 2005 to 2016 and although it has since declined from this historic high (11.8 million), it remains 19% above the long-term average of 7.9 million [24]. In contrast, the mallard population in the Great Lakes portion of the mid-continent area was about 28% lower from 2006 to 2020 (mean = 0.72 million) than from 1995 to 2005 (mean = 1.00 million) [24]. Prevalence of game-farm genes in mallards does decrease moving westward, but game-farm and hybrid mallards still made up ~40% of samples of the mid-continent population [18]. More recently low rates of game-farm ancestry were detected at southern portions of the mid-continent during winter (~4%) [21], suggesting substantial regional variation in game-farm introgression. The Great Lakes region is geographically at the intersection where mallards from the mid-continent and eastern populations mix [25–27]. Consequently, we thought the Great Lakes region would have a greater proportion of mallards carrying game-farm mallard ancestry as compared to the overall mid-continent population. Declining mallard breeding abundance in the Great Lakes region is consistent with hypothesis that game-farm traits are maladaptive when they enter the wild mallard population and at least partially explain population declines in eastern North America. Here, we not only focus on describing the genetic composition of mallards in the Great Lakes region, but link genetic ancestry with geographical breeding origins through isotopic analyses.

We focused on determining the genetic composition and natal origins of mallards migrating through the Great Lakes region by analyzing mallards harvested at private-marsh complexes in northwestern Ohio during the 2019 autumn hunting season. We assayed thousands of nuclear loci and assigned genetic ancestry across samples, enabling us to determine the proportions comprised of wild, game-farm, and filial classes of game-farm × wild hybrids (e.g., F1, F2, etc.) of our sample. We also assigned these mallards to geographic natal origins using the ratio of stable isotopes of hydrogen (^2^H/^1^H) from feathers (hereafter *δ*^2^H_f_) grown on natal areas because these isotopes produce a strong, predictable latitudinal gradient across broad geographic regions [28–31]. Unlike band-recovery data that can be spatially limited by accessibility and not representative of origins if juvenile mallards are not banded before they could fly [25, 32], a stable isotope approach decreases spatial biases associated with specific banding locations because sampling of metabolically inert flight feathers grown during development can be used to link ducks to remote areas of origin [33]. We sampled hatch-year (HY) mallards because they grow feathers on breeding grounds prior to fledging. As such, we were able to estimate breeding locations where game-farm derived genetic variation may be more likely to originate, and their proportional contributions to the mallard population migrating through an important stop-over location in the Great Lakes region. Mallards using the marshes of northwestern Ohio during their autumn-winter migration originate from mid-continent and eastern breeding populations [25–27]. Consequently, mallards harvested in this region provided a random sampling opportunity of individuals from much of the eastern half of North America during their autumn-winter migration. Similar to Palumbo et al. [34], we also determined percentages of mallards that could be classified as being produced locally (i.e., ≤ 1 standard deviation in *δ*^2^H_f_ of our sampling locations) and those with an isotopic signature of an immigrant to determine the extent that we sampled mallards from a broad geographic area. We predicted that pure wild mallards would originate from farther north and west (i.e., mid-continent population) than game-farm admixed individuals that primarily exist farther east in North America. We also predicted more northern mallards with increasingly wild genetic ancestry and respective stable hydrogen isotopic values to be present in later months of our sampling period as they migrated through and became increasingly available for harvest.

## Materials and methods

### Sample collection

We obtained HY mallards that were legally harvested at private-hunting clubs in Ottawa and Sandusky counties in northwestern Ohio during waterfowl hunting season, 4 October–19 December 2019. Use of animals in this study followed all federal and state guidelines regarding the ethics of animal welfare. All ducks were aged and sexed using plumage characteristics [35]. We sampled wing muscle and P1 (i.e., the primary feather adjacent to the secondaries) from each mallard for molecular and isotopic analyses, respectively.

### DNA extraction, ddRAD-seq libraries, sequencing, and bioinformatics

Genomic DNA was extracted from 296 mallards using and following manufacturer’s protocols for the DNeasy Blood and Tissue kit (Qiagen, Valencia, CA, USA). DNA quality was ensured based on the presence of high molecular weight bands visualized using gel electrophoresis with a 1% agarose gel, and quantified DNA concentration using a Qubit 3 Flourometer (Invitrogen, Carlsbad, CA) to ensure a minimum concentration of 20 ng/μL. Following, double-digestion we constructed restriction-site associated DNA sequencing (ddRAD-seq) fragment libraries following Lavretsky et al. [36], but with fragment size selection for average fragment lengths of 350 base-pairs followed Hernandez et al. [37]. In short, genomic DNA was enzymatically fragmented using SbfI and EcoRI restriction enzymes (New England Biolabs, Ipswich, MA), and Illumina TruSeq (Illumina, San Diego, CA) compatible barcodes ligated, permitting future de-multiplexing. Samples were pooled in equimolar concentrations and sent for 150 base pair, single-end sequencing on an Illumina HiSeq X with Novogenetics (Sacramento, CA, USA). Raw Illumina reads were deposited in the National Center for Biotechnology Information’s Sequence Read Archive (SRA; http://www.ncbi.nlm.nih.gov/sra; BioProject *TBD*).

We de-multiplexed raw Illumina reads using the *ddRADparser*.*py* script of the BU ddRAD-seq pipeline [38] based on perfect barcode/index matches. We note that previously published ddRAD raw sequence data generated using the same protocols as described above for other wild mallards [39] and game-farm mallards [19] were also included in analyses, serving as references in molecular analyses. Bioinformatics followed Lavretsky et al. [19] with all steps through genotyping automated using a custom in-house Python script (Python scripts available at https://github.com/jonmohl/PopGen). We first trimmed or discarded poor quality sequences using trimmomatic [40] with the remaining quality reads aligned to a reference mallard genome [2] using the Burrows Wheeler Aligner v. 07.15 (bwa; [41]). Next, samples were sorted and indexed in Samtools v. 1.6 [42] and combined using the “mpileup” function with the following parameters “-c–A -Q 30 -q 30,” which set a base pair and overall sequence PHRED score of ≥30 to ensure that only high-quality sequences are retained. Finally, we further filtered for any base-pair missing >5% of samples, which also required a minimum base-pair sequencing depth coverage of 5X (i.e., 10X per genotype) and quality per base PHRED scores of ≥30 using VCFtools v. 0.115 [43].

Though only autosomal loci were used in subsequent population structure analyses, the alignment of ddRAD-seq loci to our reference genome also provided positions across sex chromosomes, allowing us to use differences in expected sequencing depth to demarcate sex across samples. The heterogametic sex (i.e., female; ZW) is expected to have half the sequencing depth across both sex-chromosomes as compared to autosome-linked loci, whereas the homogametic sex (i.e., males; ZZ) showing the same sequencing depth at Z-sex chromosome and autosome linked loci and no W-linked loci (also see [36]). Raw Illumina reads were deposited in the National Center for Biotechnology Information’s Sequence Read Archive (SRA; http://www.ncbi.nlm.nih.gov/sra; BioProject PRJNA911314; See S1 Table for sample specific accession numbers).

### Population structure

Population structure was evaluated using autosomal ddRAD-seq bi-allelic single nucleotide polymorphisms (SNPs) only. Prior to analyses, datasets were filtered for singletons (i.e., minimum allele frequency [maf] = 0.0023) and any SNP missing ≥5% of data across samples using PLINK v. 0.70 [44]. Independence between SNPs was based on pair-wise analysis of linkage disequilibrium (LD) (—indep-pairwise 2 1 0.5) in which 1 of 2 linked SNPs are randomly excluded if an LD correlation factor (*r*^2^) > 0.5 was obtained. All analyses were done without *a priori* information on population or species identity.

Population structure was visualized using the PCA function in PLINK to perform a principal component analysis (PCA). Next, we used ADMIXTURE version 1.3 [45, 46] to attain maximum likelihood estimates of population assignments for each individual, following dataset preparation steps as outlined in Alexander et al. [47]. ADMIXTURE was run with a 10-fold cross validation, incorporating a quasi-Newton algorithm to accelerate convergence [48]. Each analysis used a block relaxation algorithm for point estimation and terminated once the change in the log-likelihood of the point estimations increased by <0.0001. Each analysis was run for *K* populations of 1 through 5, and with 100 iterations per each value of *K*. The optimum *K* in each analysis was based on the average of cross-validation errors across the iterations per *K* value; however, we examined additional values of *K* to test for further structural resolution across analyses. We used the R package PopHelper [49] to convert ADMIXTURE outputs into CLUMPP input files at each *K* value, and to determine the robustness of assignments of individuals to populations at each *K* value with the program CLUMPP version 1.1 [50]. In CLUMPP, we employed the Large Greedy algorithm and 1,000 random permutations. Final admixture proportions for each *K* value and per sample assignment probabilities (Q estimates; the log likelihood of group assignment) were based on CLUMPP analyses of all 100 replicates per *K* value.

### Establishing hybrid indices and hybrid assignment

To assess the effectiveness of our molecular dataset in distinguishing between classes of generational hybrids, and to more directly assign putative hybrids to those classes, we simulated expected assignment probabilities with our empirical data for first generation hybrids (F1) and 9 generations of backcrosses (F2–F10) based on methods outlined in Lavretsky et al. [51]. We used the same ddRAD-seq bi-allelic nuclear SNP set analyzed for population structure in simulations, but only including reference wild mallard and game-farm mallards. We first generated 10 F1 hybrids by randomly sampling an allele from the wild mallard and game-farm mallard gene pools across bi-allelic SNP positions; we randomly sampled each position based on a probability proportional to the allelic frequency in each respective gene pool. We then backcrossed 5 hybrids to the parental gene pool for 9 generations. Following, we ran 10 independent simulations. Simulations were then combined with the original reference set and inputted into ADMIXTURE to estimate assignment probabilities for a *K* of 2; which was the optimum *K* population value (see results). We ran 25 iterations for each of the 10 simulated datasets. Subsequently, we combined all 250 ADMIXTURE outputs and converted in PopHelper into CLUMPP input files. We employed the Large Greedy algorithm and 1,000 random permutations with final admixture proportions for each *K* and per sample assignment probabilities based on CLUMPP analyses of all 250 replicates per *K*. Per generation expected assignment probabilities were based on the average of all 10 (F1) or each of the 5 (F2–F10) backcrosses, along with each lower and upper limit.

### Hydrogen stable isotope analysis

We sent feather samples for *δ*^2^H analysis at the Cornell Stable Isotope Laboratory in Ithaca, NY. After washing in a 2:1 chloroform:methanol solution, feather samples were air dried under a fume hood and a subsample (0.35 mg) of vane material was loaded into silver capsules, crushed, and placed with internal lab standards into a desiccator for a minimum of three days prior to analysis. Samples were then loaded into a Zero Blank carousel under helium flow. Pyrolysis combustion on glassy carbon was at 1350°C in a Thermo Scientific Temperature Conversion Elemental Analyzer (TC/EA; Bremen, Germany) coupled via a Conflo IV (Thermo Scientific) to a Thermo Scientific Delta V Advantage isotope ratio mass spectrometer. Analysis of *δ*^2^H was conducted using the comparative equilibration method of Wassenaar and Hobson [52] with 2 calibrated keratin reference materials (CBS, *δ*^2^H = -197‰; KHS, *δ*^2^H = -54.1‰; SPK, *δ*^2^H = -121.6‰) corrected for linear instrumental drift. Based on within-run analyses of a third keratin standard, measurement error was approximately ± 3‰ for hydrogen isotopes in feather (*δ*^2^H_*f*_). All *δ*^2^H values are reported relative to the Vienna Standard Mean Ocean Water–Standard Light Antarctic Precipitation scale.

### Assignment to geographic origin using stable isotopes

To estimate natal origins of harvested birds, we used likelihood-based algorithms to produce spatially explicit assignments of source areas based on analysis of feather *δ*^2^H data [53, 54]. Similar to prior studies [34, 55–57], we created a map (hereafter isoscape) by applying a rescaling function derived by regressing δ^2^H_f_ of known-origin mallards against amount-weighted growing-season average precipitation (δ^2^H_p_) [58] (δ^2^H_f_ = -27.4 + 1.28 * δ^2^H_p_). We acquired the breeding range of mallards from BirdLife International [58], and the spatially explicit assignments were restricted (i.e., masked) to only those areas of the continent occurring in this range. Finally, mallards originating from outside the mid-continent and eastern populations made up < 1% of direct band recoveries (i.e., recoveries in the hunting season following banding) in Ottawa and Sandusky counties of Ohio, so we clipped the breeding range to remove the western breeding range (Fig 1). Additionally, to eliminate erroneous assignments, we clipped the breeding range along the 100^th^ meridian because precipitation regimes in the western U.S. interfere with longitudinal *δ*^2^H_p_ gradients. We estimated the likelihood that individual cells (i.e., pixels) within the isoscape represented a potential source area for a given sample by comparing the observed *δ*^2^H_f_ against the isoscape predicted *δ*^2^H_f_ using a normal probability density function [34, 53]. We then applied Bayes’ Theorem to assess the posterior probability that an individual pixel within the isoscape was the putative source area of a given sample [34, 53]. After normalizing the probabilities to sum to one, we assigned individuals to source areas within the isoscape by selecting the raster cells that were consistent with the upper 67% of estimated probabilities of origin for each individual and coded those as one and all others as zero, consistent with 2:1 odds. We subsequently summed the results of the assignments over all individuals by addition of the surfaces to attain a final surface of the distribution in the number of individuals assigned to each pixel [34, 53].

In addition to assigning birds to their natal origins, we classified birds as locals versus immigrants using likelihood-based assignments [34]. We defined local birds as any individual whose *δ*^2^H_f_ was consistent with the expected value for birds (*δ*^2^H_fd_) originating in Ottawa and Sandusky counties, Ohio. Using the rescaled δ^2^H_p_, we predicted the mean *δ*^2^H_fd_ to be -82.5‰ to -84.5‰. We used a normal probability density function to compare the observed *δ*^2^H_f_ for unknown-origin birds against the predicted mean *δ*^2^H_fd_ to assess the likelihood that a given bird had grown its feathers in Ottawa or Sandusky counties of Ohio, given expected variation in *δ*^2^H_f_ [34]. If *δ*^2^H_f_ was within 12.8‰ (1 standard deviation) of the expected *δ*^2^H_f_ for Ottawa and Sandusky counties of Ohio (*δ*^2^H_fd_), we classified individual ducks as locals; others were classified as immigrants.

### Weighting isotope results with band recoveries

Stable hydrogen isotopes (i.e., *δ*^2^H_p_) produce a predictable latitudinal gradient but provide little information longitudinally [53]. Thus, we weighted isotope results by applying a band recovery distribution from the eastern and mid-continent populations. To do so, we used available band recovery data to inform weighting of source population origination to the mid-continent or eastern populations. We summarized available (through 14 July 2021) band recovery data of HY mallards from the U.S. Geological Survey Gamebirds database. The database was queried for all reported direct recoveries of HY mallards in Ottawa and Sandusky counties of Ohio, as well as for all banding stations and number of bands placed on HY mallards in the mid-continent and eastern population areas. The recovery location and their corresponding banding location were displayed in a geographic information system (hereafter GIS) (ArcGIS 10.8, Environmental Systems Research Institute Inc., Redlands, California, USA). We selected all reported recoveries through time and used their associated banding location to assign origin to each sample to the mid-continent or eastern population. A polygon encompassing banding coordinates associated with direct recoveries served as the geographic area where harvested mallards potentially originated. To determine banding effort, we extracted all banding stations and summed the number of bands placed on HY mallards in the polygon where potential direct recoveries could have originated. We then calculated the proportion of band recoveries derived from the mid-continent and eastern populations and adjusted for banding effort by dividing the total number of recovered bands by the total number of bands placed on mallards that would be available for recovery. New rasters and an assignment to origin map were then created where the pixel values within the mid-continent and eastern populations represented the banding effort informed proportion of harvest. We created assignment to origin maps for each genetic assignment (i.e., pure wild, hybrid swarm, and filials) with sufficient sample sizes.

### Statistical analyses for associating genetic assignment with geographical origin

We used linear regression models to determine if *δ*^2^H_f_ was influenced by genetic assignment (pure wild, hybrid swarm × wild, and F3), sex, and harvest date (4 October = day 0). When genetic assignment was included in the final model, we used a Tukey Honest Difference test to determine differences among means between categories at α = 0.05. We provided descriptive statistics for genetic group(s) with insufficient sample sizes to obtain statistical significance (see results).

## Results

### Sequencing and molecular data output

A total of 2,951 ddRAD-seq autosomal loci (692,460 base-pairs (bp)) were recovered across samples that included 2,751 autosomal (658,008 bp), 193 Z-sex chromosome linked (33,509 bp), and 7 W-sex chromosome linked (943 bp) ddRAD-seq loci that met our criteria for sequencing coverage and missing data. We attained an average depth of 118 sequences/locus/sample (depth range 18–163 sequences/locus/sample). Moreover, we confirmed sex identity across samples by plotting sequencing depth ratios between autosomal and recovered W- and Z-sex chromosome linked ddRAD-seq loci (S1 Fig). Finally, we attained 593 base-pairs of the mtDNA control region across all samples.

### Nuclear simulations and population structure

**Fig 2:**
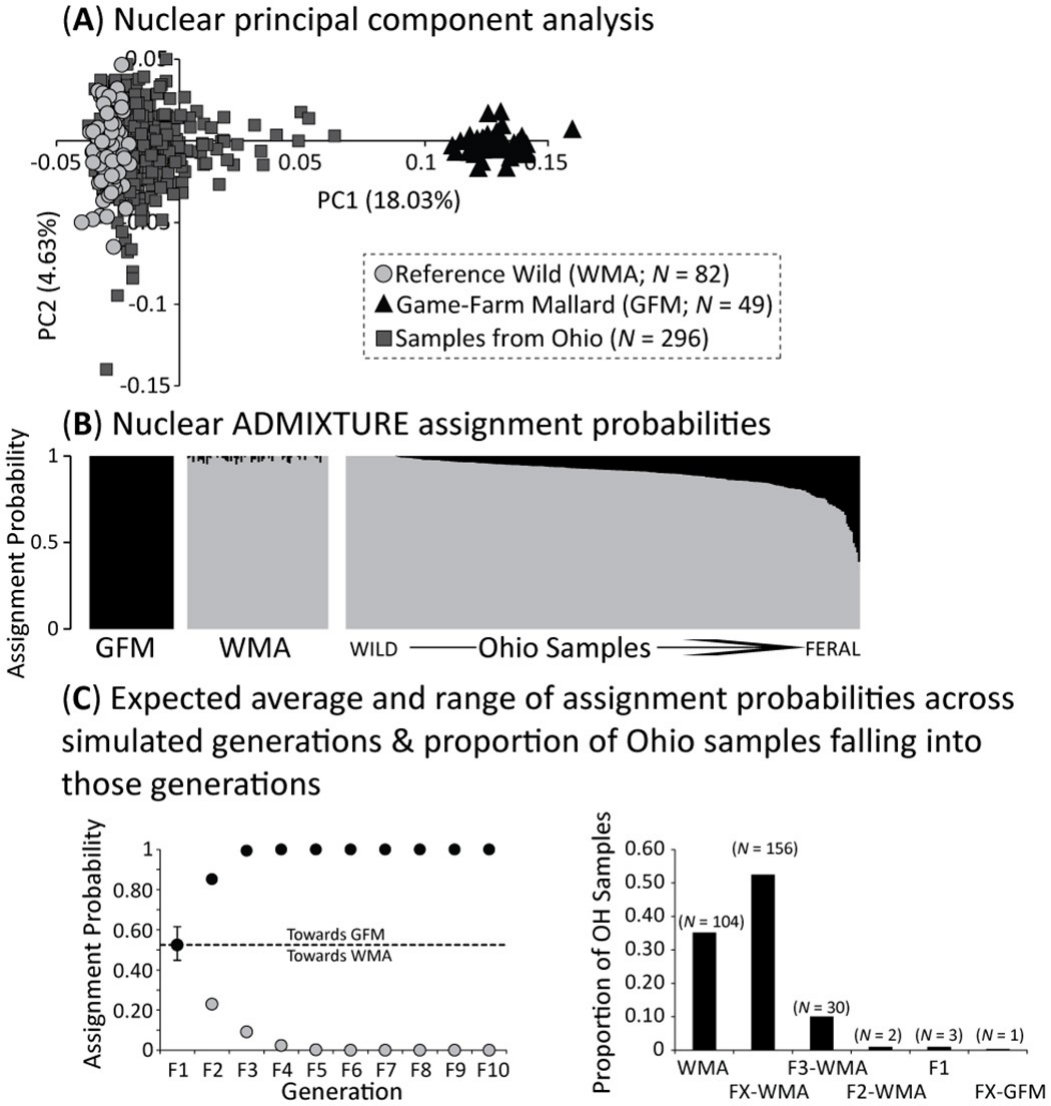
Population structure analyses based on 22,136 independent bi-allelic ddRAD-seq SNPs assayed across Ohio (OH) samples, as well as reference wild (WMA) and game-farm (GFM) mallards, including (A) a plot of the first two principal components of the principal components analysis (PCA) and (B) ADMIXTURE assignment probabilities attained under an optimum *K* populations of 2 (S2B Fig). Finally, we provide the (C) expected average and range of assignment probabilities across simulated generations (left), as well as the proportion of Ohio samples falling into those generations (right).

A total of 22,136 (of 22,139) independent bi-allelic ddRAD-seq SNPs were retained for nuclear population structure analyses. Plotting the first two principal components explained a total of 22.66% of the variance (S2A Fig), and which separated reference wild and game-farm mallards with Ohio samples scattered from the wild reference mallard set towards game-farm mallards (Fig 2A). Plotting ADMIXTURE assignment probabilities under the optimum *K* population of two (S2B Fig) distinguished between wild and game-farm mallards, while Ohio samples possessed assignment probabilities to either the wild mallard genetic cluster only or had assignments to both clusters (Fig 2B).

Formal assignment of samples of admixed ancestry were based on hybrid filial class cut-offs established through our simulations that were run under a *K* population model of two in ADMIXTURE (Fig 2C). First, we designated any sample within the assignment probabilities range recovered among reference wild mallard set as pure wild mallards, which included any sample with ≤5% alternative genetic cluster assignment. For the remaining admixed samples, we were able to reliably identify between F1 hybrids, as well as F2 and F3 backcrossed generations (S2 Table). Note that any sample with mixed ancestry assignments intermediate to those recovered in simulated F1–F3 generations represented backcrosses of unknown age (e.g., FX hybrid × hybrid crosses, parental hybridization switching between generations; [18]; and were designated as either backcrossed with more wild (i.e., FX-WMA) or game-farm (i.e., FX-GFM) mallard, respectively (Fig 2C). Categorization of samples based on simulations recovered 35% (*n* = 104) of Ohio samples as pure wild mallard, while the majority (53%; *n* = 156) being within the FX-WMA category (Fig 2C). A total of three (1%), two (0.7%), and 30 (10%) of Ohio samples were designated as F1, F2-WMA, and F3-WMA, respectively (S1 Table). No Ohio sample was found to be game-farm mallard (i.e., ≥ 95% to the game-farm mallard genetic cluster), but one sample was designated as a hybrid backcross towards game-farm mallard (i.e., FX-GFM; Fig 2C; S1 Table).

### Stable isotope results for local vs. migrant

We analyzed 296 HY mallards of unknown origin and classified 24% (*n* = 71) of individuals as local whose *δ*^*2*^H_f_ was consistent with feather growth in Ottawa or Sandusky counties of Ohio, and 76% (*n* = 225) of individuals as immigrants whose *δ*^*2*^H_f_ was consistent with feather growth outside these counties.

### Relationship of stable isotopes and genetics

**Fig 3:**
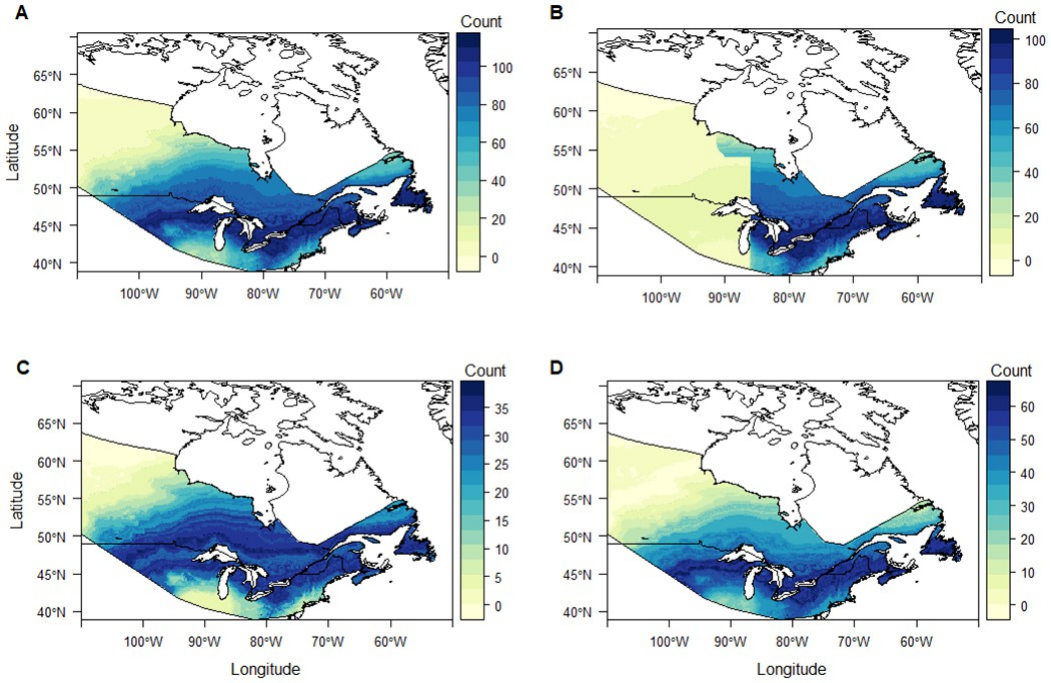
Geographic depiction of probable origins of hatch-year mallards harvested in Ottawa and Sandusky counties, Ohio, USA, from October to December 2019. Legend numbers correspond to the number of individuals assigned to each pixel based on a 2:1 odds ratio. A) all samples in our study without application of a banding data informed weighted spatial mask, B) origins constrained by a banding data informed weighted spatial mask, C) Origins of pure wild mallards without application of a banding data informed weighted spatial mask, and D) Origins of hybrid-swarm mallards without application of a banding data informed weighted spatial mask. U.S. and Canadian political boundaries were obtained through the R package ‘rnaturalearth’. Atlantic and Mississippi flyway shapefiles were obtained from the metadata of Weltzin et al. 2018 (https://www.sciencebase.gov/catalog/item/5b75d10ee4b0f5d5787fea6f). Atlantic and Mississippi flyway boundaries and data were used from Weltzin et al. 2018 (https://www.sciencebase.gov/catalog/item/5b75d10ee4b0f5d5787fea6f). The calibration equation used to produce the assignment to origin was obtained from van Dijk et al. 2014. Except for the shapefiles listed above, no external data was used for the creation of the figure. Figures were created using the R statistical framework (version 4.0.2, R Core Team 2018).

We detected that *δ*^*2*^H_f_ values were explained by genetic assignment (F_2, 286_ = 8.80, *P* < 0.01) and harvest date (F_1, 286_ = 8.33, *P* < 0.01) (Table 1), but not sex (*P* = 0.25). Model predicted *δ*^2^H_f_ changed by -16.6‰ from 4 October to 19 December; approximately the northern distance from our northwestern Ohio study area to Port Austin, MI, ~288 km or 2.5° latitude. Among genetic assignments, we detected that pure wild mallards (*n* = 104) had more negative (i.e., more northern) *δ*^*2*^H_f_ values (*P* < 0.01; -121.05 ± 2.43 [SE]) than hybrid swarm mallards (-108.23 ± 1.99; *n* = 156), but F3 (-113.33 ± 4.53; *n* = 30) did not differ from pure wild or hybrid swarm mallards (*P* ≥ 0.29; Table 1, Fig 3). Values for *δ*^*2*^H_f_ from genetic groups with insufficient samples sizes for statistical testing included, F1 (n = 3, mean = -105.86 ± 2.80, min = -110.76, max = -101.06), F2 (n = 2, min = -111.64, max = -106.91), and hybrid × game-farm (n = 1, -99.46).

**Table 1:** Parameter estimates ± standard error (SE) and 95% confidence limits (CL) of factors affecting *δ*^2^H_f_ of hatch-year mallards (*Anas platyrhynchos*) harvested in northwestern Ohio, 4 October–19 December 2019.

Parameter_df_	β ± SE	95% CL
Intercept	-113.26 ± 3.61	-120.35 to -106.16
Date_1, 287_	-0.22 ± 0.07	-0.36 to -0.07
Genomics_2, 287_		
F3	7.72 ± 5.15	-2.41 to 17.85
Hybrid swarm mallard	12.82 ± 3.14	6.64 to 19.01
Pure wild mallard	0.00	0.00

## Discussion

Studies of mallard genetics have become increasingly common because of concern about introgression of potentially maladaptive genes into the wild population from breeding with released game-farm mallards [17, 19]. Most releases of game-farm mallards in North America have occurred on the United States portion of the Atlantic coast (USFWS 2003). Across broad regions, estimates are that ~92% of mallards in Atlantic coast states and ~40% in the mid-continent had substantial (> 10% assignment probabilities) game-farm mallard derived ancestry in 2010 [18]. In more western locales of North America only 3% of mallards possessed substantial game-farm mallard ancestry [18]. Similarly, at southern latitudes in the mid-continent only ~4% of samples were game-farm hybrids in 2021 [21]. We detected that 65% of sampled mallards had substantive amounts of game-farm genetic ancestry at our Ohio study area. We posit that the greater hybrid prevalence in Ohio was from movement of eastern mallards into the Great Lakes region on their way to more southern locales. Differences between our results and those of Davis et al. [21] suggest differences in migration and wintering areas for mid-continent and eastern population mallards. Moreover, hybrids reported by Davis et al. [21] in southern parts of the mid-continent were late backcrosses as compared to the wide variance of hybrid ancestry detected in the current study (Fig 2B). We suspect this regional variation in percentage of mallards with game-farm genes exists because those with greater percentage of game-farm ancestry are less likely to migrate to traditional wintering areas at southern latitudes of North America because they lack this innate behavior. Combined, results provide evidence of meta-population dynamics in the mid-continent that will require greater efforts coupling genetics and movement ecology studies to understand variation in migration patterns among mallard genotypes.

Nearly all possible genetic combinations of mallards were obtained at our study area. Most of our samples were assigned as hybrid swarm (53%) or pure wild (35%), but we also obtained three F1 hybrids (pure game-farm × pure wild cross), which can only result from annual interbreeding between game-farm and wild mallards. We conclude that continued game-farm introgression into the wild mallard population is not isolated to the eastern population of mallards in North America, and may be increasing and more widespread than previously reported. More intensive and geographically broad sampling of the mid-continent region will be required to better understand whether the apparent ongoing interbreeding between game-farm and wild mallards is due to local release efforts or immigrants from the eastern population of mallards. Additionally, a thorough survey of the number and locations of game-farm releases throughout North America would help refine our understanding of the spatial potential of game-farm mallard introgression.

Pure wild mallards in our sample had *δ*^2^H_f_ values that indicated they geographically originated from farther north than hybrid swarm mallards. Stable hydrogen isotopes (i.e., *δ*^2^H_p_) produce a predictable latitudinal gradient, but provide little information longitudinally [59]. We used weighting of the isotope results by applying a band recovery distribution from the eastern and mid-continent populations, which weighted a greater portion of our sample longitudinally towards the eastern population. However, 17% of our samples had *δ*^2^H_f_ < -140.0, which could only have originated from the mid-continent population (i.e., prairie, parkland, or boreal) or from areas north of the applied mallard range in Quebec. As such our weighted origin maps should be interpreted with caution because they can introduce error from the uneven spatial distribution or lack of banding effort in regions where mallards originated. Further, although not significant, mean *δ*^2^H_f_ from F1 through F3 hybrids in our study followed a predictable pattern consistent with individuals possessing greater levels of game-farm genetic assignment originating at more southern latitudes (i.e., F1) and future hybrid filial generations occurring in more northern locales (i.e., F2 through F3). For mallards with *δ*^2^H_f_ < -140.0, most were wild (55%), 35% were hybrid swarm, and 10% were F3 which is consistent with introgression of game-farm genetic variation moving north and west in a generational pattern consistent with breeding ecology of dabbling ducks whereby males follow females to breeding areas after pairing during the non-breeding period [9]. We speculate that we did not detect differences in *δ*^2^H_f_ between hybrid filials and other genetic categories because of small sample sizes. Our results suggest game-farm mallard introgression into the mid-continent population of mallards in the prairie, parkland, and boreal regions. This could be cause for concern because other areas with widespread introgression of game-farm genes have seen declining mallard breeding abundance [22, 23].

Mallards are considered facultative migrants [9], whereby they typically migrate when weather or deteriorating environmental conditions make them move during autumn migration [60, 61]. Consistent with this pattern, we detected that mallards originating from more northern latitudes were harvested later in the duck hunting season in northwestern Ohio. Further, our result that 76% of harvested mallards in northwestern Ohio were classified as immigrants is similar to findings by Palumbo et al. [34] from Lake St. Clair, Ontario, approximately 100km north of our study area. These results contrast with a sample of 1,254 HY mallards from throughout the Atlantic coast states that indicated that origin, as determined by *δ*^2^H_f_, was unrelated to timing of harvest [62, 63]. Differences between our northwestern Ohio study site and those from farther east could be related to differences in genetic composition of mallards between these regions, whereby seasonal movement patterns differ because eastern population mallards have greater genetic input from game-farm mallards. Wild mallards may be adapted to migrate when weather conditions deteriorate and move facultatively towards ancestral wintering grounds [9, 60]. In contrast, mallards bred through artificial selection on game-farms may not have a similar phenotypic response to migrate, which may produce less predictable seasonal movements [64]. Early studies during a time of large releases of game-farm mallards suggest failure of these ducks to migrate similar to wild congeners [65], but the hypothesis that percentage of game-farm genes influences migratory behavior needs further testing. Knowing the genetics of a banded sample of mallards or tracked them by telemetry would aid in our understanding of migratory movements of different genotypes of mallards.

We found that a surprisingly high proportion of HY mallards harvested in northwestern Ohio had some form of hybridization with game-farm mallards and that pure wild mallards originated from areas farther north (and also likely west) compared to hybrid, admixed individuals. Our findings add to growing concern that continued and possibly increasing introgression of game-farm mallard genes into the wild mallard breeding population may be causing population declines in some regions, particularly in the U.S. portion of eastern North America. We advocate for expanded use of stable feather isotopes in combination with genomic methods, as we have done here, to aid in disentangling the extent of the apparently negative influence of game-farm genes on the health of the North American mallard population. Specifically, focusing these efforts in the mid-continent region should be priority because of the apparent western movement of game-farm genetic variation into this mallard population that we detected. Together, coordinated efforts to collect stable isotope data, genetics, and movements of mallards would provide strong inference capacity about the distribution and effect on migratory movement of game-farm mallard genetic variation across North America.

## Supporting information

S1 Table Raw genetic, isotope, and additional metadata from hatch-year mallards (*Anas platyrhynchos*) harvested in northwestern Ohio, 4 October–19 December 2019. (XLSX)

S2 Table Simulated expected average, minimum, and maximum assignment probability values to wild mallard (WMA) genetic cluster based on ddRAD-seq autosomal bi-allelic SNPs for 10 hybrid classes determined between wild and game-farm (GFM) mallards. The parental backcross for each generation is provided, and those generations that are genetically distinguishable denoted.
(PNG)

S1 Fig Plot of Z- or W-Sex chromosome versus autosomal (A) sequencing depth to identify sex across samples. (TIF)

S2 Fig (A) Proportion of variation explained by each principal components of the principal components analysis (PCA; Fig 1A). (B) CV-Error values averaged across the 100 ADMIXTURE analysis replicates for each of the evaluated *K* population values of 1–5; the optimum *K* population of two is denoted.
(TIF)
