# Supplementary figures and images for: Population genetics and geographic origins of mallards harvested in northwestern Ohio
Source: PLoS One. 2023 Mar 15;18(3):e0282874. doi: 10.1371/journal.pone.0282874 (PMC10016643; doi:10.1371/journal.pone.0282874)

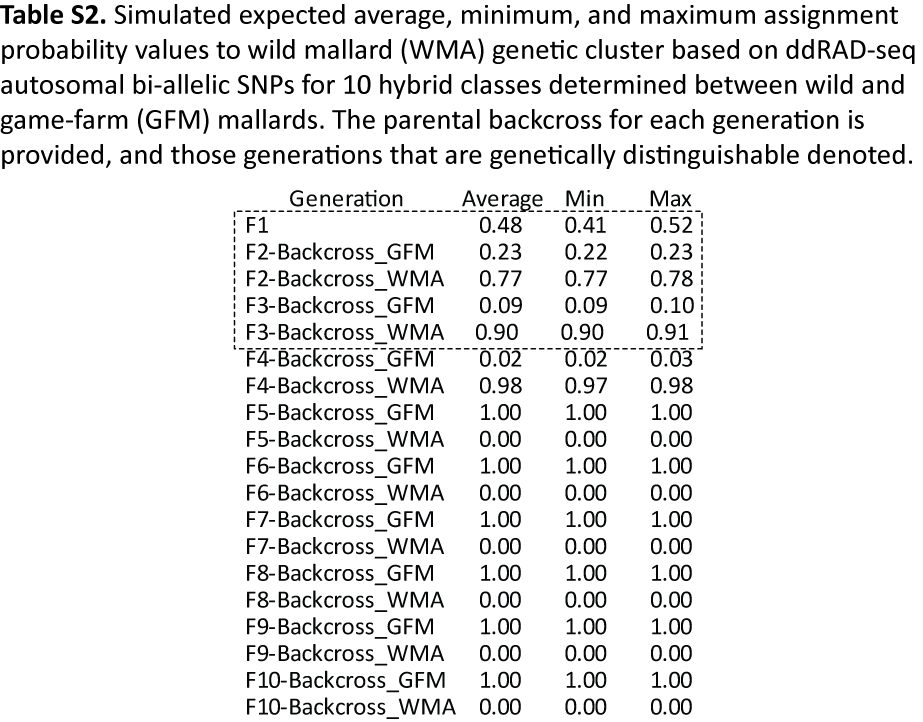

Supplement: S2 Table — The parental backcross for each generation is provided, and those generations that are genetically distinguishable denoted. (PNG) [file pone.0282874.s002.png]

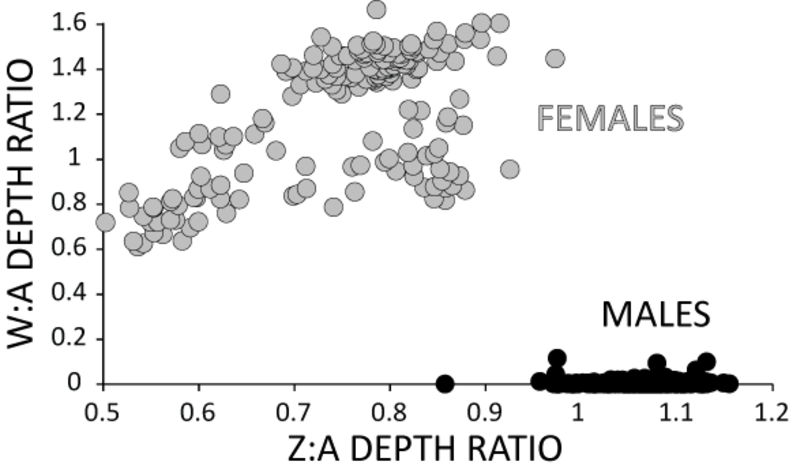

Supplement: S1 Fig — (TIF) [file pone.0282874.s003.tif]

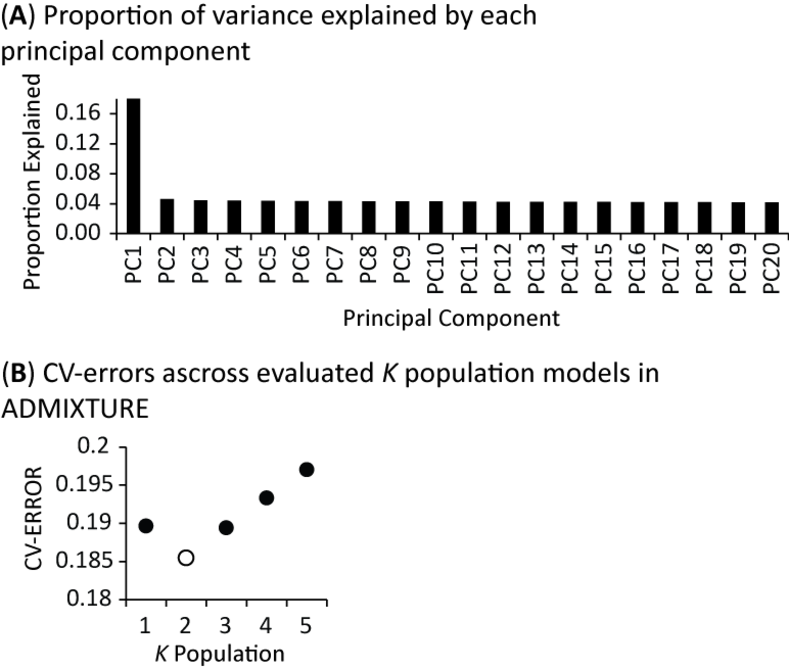

Supplement: S2 Fig — (A) Proportion of variation explained by each principal components of the principal components analysis (PCA; Fig 1A). (B) CV-Error values averaged across the 100 ADMIXTURE analysis replicates for each of the evaluated K population values of 1–5; the optimum K population of two is denoted. (TIF) [file pone.0282874.s004.tif]
